# High Polyhydroxybutyrate Production in *Pseudomonas extremaustralis* Is Associated with Differential Expression of Horizontally Acquired and Core Genome Polyhydroxyalkanoate Synthase Genes

**DOI:** 10.1371/journal.pone.0098873

**Published:** 2014-06-02

**Authors:** Mariela V. Catone, Jimena A. Ruiz, Mildred Castellanos, Daniel Segura, Guadalupe Espin, Nancy I. López

**Affiliations:** 1 Departamento de Química Biológica, Facultad de Ciencias Exactas y Naturales, Universidad de Buenos Aires, Buenos Aires, Argentina; 2 Instituto de Investigaciones en Biociencias Agrícolas y Ambientales, CONICET, Buenos Aires, Argentina; 3 Departamento de Microbiología Molecular, Instituto de Biotecnología, Universidad Nacional Autónoma de México, Cuernavaca, Morelos, México; 4 IQUIBICEN, CONICET, Buenos Aires, Argentina; University of North Dakota, United States of America

## Abstract

*Pseudomonas extremaustralis* produces mainly polyhydroxybutyrate (PHB), a short chain length polyhydroxyalkanoate (sclPHA) infrequently found in *Pseudomonas* species. Previous studies with this strain demonstrated that PHB genes are located in a genomic island. In this work, the analysis of the genome of *P. extremaustralis* revealed the presence of another PHB cluster *phbFPX,* with high similarity to genes belonging to *Burkholderiales,* and also a cluster, *phaC1ZC2D,* coding for medium chain length PHA production (mclPHA). All mclPHA genes showed high similarity to genes from *Pseudomonas* species and interestingly, this cluster also showed a natural insertion of seven ORFs not related to mclPHA metabolism. Besides PHB, *P. extremaustralis* is able to produce mclPHA although in minor amounts. Complementation analysis demonstrated that both mclPHA synthases, PhaC1 and PhaC2, were functional. RT-qPCR analysis showed different levels of expression for the PHB synthase, *phbC*, and the mclPHA synthases. The expression level of *phbC*, was significantly higher than the obtained for *phaC1* and *phaC2*, in late exponential phase cultures. The analysis of the proteins bound to the PHA granules showed the presence of PhbC and PhaC1, whilst PhaC2 could not be detected. In addition, two phasin like proteins (PhbP and PhaI) associated with the production of scl and mcl PHAs, respectively, were detected. The results of this work show the high efficiency of a foreign gene (*phbC*) in comparison with the mclPHA core genome genes (*phaC1* and *phaC2*) indicating that the ability of *P. extremaustralis* to produce high amounts of PHB could be explained by the different expression levels of the genes encoding the scl and mcl PHA synthases.

## Introduction

Polyhydroxyalkanoates (PHAs) are reserve polymers produced by a wide range of bacteria accumulated in intracellular granules as carbon and energy storage material under unbalanced growth conditions. These polymers exhibit thermoplastic and elastomeric features in addition to other interesting properties, such us biodegradability and biocompatibility, which make them promising industrial materials [1]. The physical and chemical properties of these bioplastics are directly related to the monomer composition of the polymer. According to the number of carbons in the monomer units, PHAs can be classified into two classes: short-chain-length PHAs (sclPHAs) and medium-chain-length PHAs (mclPHAs). The sclPHAs, composed of C3 to C5 monomer units, are thermoplastic in nature, hard and brittle, meanwhile mclPHAs, which are formed of C6 to C14 monomer units, are elastomeric in nature.

The gene cluster involved in polyhydroxybutyrate (PHB) production, the most widely produced sclPHA by bacteria, includes three structural genes named *phaA* or *phbA*, *phaB* or *phbB* and *phaC* or *phbC*, encoding a β-ketothiolase, a (R)-specific acetoacetyl-CoA reductase and a class I synthase, respectively. Class I synthase is the key enzyme for PHB biosynthesis.

The mclPHA cluster (*phaC1ZC2DFI*) is well conserved among the mclPHA producers bacteria. This cluster encode two class II synthase genes (*phaC1* and *phaC2*) separated by a gene encoding a PHA depolymerase (*phaZ*); a transcriptional activator (*phaD*) and two genes (*phaFI*) encoding phasin-like proteins [2]. In most *Pseudomonas,* β-oxidation and fatty acid *de novo* synthesis are used to convert fatty acid or carbohydrate intermediates, respectively into (R)-3-hydroxyacyl-CoAs, which are used as substrates by the Class II PHA synthases [3].

Synthesis of mcl-PHAs has been observed in fluorescent and non-fluorescent *Pseudomonas* species, whereas PHB production is not a common characteristic of *Pseudomonas* spp. [4]. PHB production has been reported in some *Pseudomonas* strains, such as those included in the “*P. oleovorans* group” [5].

*Pseudomonas extremaustralis* is a highly stress resistant bacterium isolated from Antarctica, able to accumulate large quantities of PHB [6]. Previous studies in this strain have allowed the identification of a PHB gene cluster (*phaRBAC*) located within a genomic island [7], [8]. The PHB gene cluster of *P. extremaustralis* showed a mosaic structure with genes of different origin, indicating their acquisition by horizontal transfer [8]. Recently, we have obtained the complete genome of *P. extremaustralis* 14-3b (DSM 25547), a natural derivative of the type strain (14-3^T^) [9]. This strain accumulates mainly PHB and minor amounts of mclPHAs when grown with octanoate or glucose as carbon source.

Based on this evidence, we hypothesized that genes involved in mclPHA production should be also present in the genome of *P. extremaustralis.* In the present work, we identified and analyzed the genes involved in mclPHA metabolism in *P. extremaustralis.* Besides, the PHB and mclPHA content as well as the expression of the genes encoding the corresponding synthases were compared under different growth conditions. Finally, the PHA granules of *P. extremaustralis* were isolated in order to identify the proteins bound to PHA in this bacterium.

## Materials and Methods

### Bacterial strains, plasmids and growth conditions

The bacterial strains and plasmids used in this study are listed in Table 1. Bacteria belonging to the *Pseudomonas* genus were cultured at 30°C in 0.5NE2 medium [10] supplemented with sodium octanoate (0.25%) or glucose (0.27 or 3%) as carbon source. The 0.5NE2 medium has reduced nitrogen content that favors PHA accumulation [10]. *Escherichia coli* was cultured at 37°C in Luria Broth (LB). When necessary, antibiotics were added at the following concentrations: 50 µg.ml^−1^ kanamycin (Km), 100 µg.ml^−1^ ampicillin (Amp), 10 µg.ml^−1^ gentamicin (Gm) and 10 µg.ml^−1^ tetracycline (Tet).

**Table 1:** Bacterial strains and plasmids used in this study.

Strains and plasmids	Relevant characteristics	Source or references
Bacterial strains		
*P. extremaustralis* DSM 25547 (14-3b)	Wild type PHB producer	[9]
*P. putida* GPp104	PHA negative mutant	[10]
		
*Escherichia coli* DH5α	*fhuA2* Δ*(argF-lacZ)U169 phoA glnV44 Φ80* Δ*(lacZ)M15 gyrA96 recA1 relA1 endA1 thi-1 hsdR17^−^*	[11]
*E. coli* S17-1	Conjugative. *recA*, *tra* genes	[12]
Plasmids		
pGEM-T Easy	Cloning vector	Promega
pBBR1MCS-5	Gm^R^; medium-copy number, conjugative	[13]
pBBR1MCS-2	Km^R^; medium-copy number, conjugative	[13]
pSJ33	Km^R^; low-copy number, conjugative	[14]
pMHZ59	pBBR1MCS-5 derivative, containing *phaC1*	This work
pMMic2	pBBR1MCS-5 derivative, containing *phaC2*	This work
pM-phbX	pBBR1MCS-2 derivative, containing *phbX*	This work
pPSJ-phbX	pSJ33 derivative, containing *phbX*	This work

### Screening of genes involved in PHA metabolism

The search of genes responsible of mclPHA production in the genome of *P. extremaustralis* was first performed using PCR amplification strategies. Degenerate primers were designed to amplify the mclPHA genes of *P. extremaustralis*, using the sequences of mclPHA genes of other *Pseudomonas* species available in the databases (Table 2). PCR amplification reactions were performed in a final volume of 50 µl containing 0.3 µM of each primer, 0.2 mM of dNTPs, and 2.5U of Go Taq polymerase (Promega). The reaction mixtures were subjected to 30 cycles of amplification in a Techne Termocycler with the following conditions: 94°C for 1 min, 50/60°C for 30 s, 72°C for 1 min and a final extension of 10 min at 72°C. Amplification products were sequenced by Macrogen Inc. (Korea). Sequences were aligned, assembled and analyzed using the following programs available online: BLAST (http://blast.ncbi.nlm.nih.gov/Blast.cgi), CAP3 Sequence Assembly Program (http://pbil.univ-lyon1.fr/cap3.php), ORFfinder (http://www.ncbi.nlm.nih.gov/projects/gorf/), and ClustalW (http://www.ebi.ac.uk/Tools/clustalw/). Promoters were predicted using Softberry program (http://linux1.softberry.com/berry.phtml).

**Table 2:** Oligonucleotides used in this study.

Gene	Sequence primer 5′ to 3′ forward (up)	Sequence primer 5′ to 3′ reverse (low)
*phaC1*	TGTTCACYTGGGAVTACC	CGGGTAATCAGGAACAGA
*phaZ*	CAAGCACACCGATTCCTGGTG	TTGAGVAGGGTGTTGGAA
*phaC2*	TGATCAACATGCGCMTGCT	GGATCCTGAACAAATACAGGGCACAT
*phaD*	CGCTTCGTGCTGTCCAAC	GCYACCAGCATCATSAYCTGRTA
*phbX*	GCGTGGAGATGAGCCAGGAAAGTTTTTTTGCGA	CGCTCGAGTCATTCGGCGTTGCCTCCCTT
*rtphaC1*	CGATGACTTGAAACGCCA	ACGGTTGCTTGATGGCTT
*rtphaC2*	GAGAAAGACCCGTGACGAAC	GCGTCCCATCTGACCGCCCAGG
*rtphbC*	CTTCGTCCTCGGATGTTCTG	ATCGACCCACCAACTCCTG
*rt16S*	CAGCTCGTGTCGTGAGATGT	AAGGGCCATGATGACTTGAC

Subsequently, the availability of the genome of *P. extremaustralis* allowed us to look for other genes involved in PHA metabolism and to determine their location using the bioinformatic tools included in the RAST server (http://rast.nmpdr.org/). Analysis of genomic islands was performed using the Island Viewer software [15].

### Complementation assays

The genes encoding the PHA synthases (*phaC1* and *phaC2*) of *P. extremaustralis* were amplified by PCR. Fragments of 2.9 and 2.1 kb containing *phaC1* and *phaC2* genes with their own promoter sequences, respectively, were cloned into the pGEM-T Easy Plasmid (Promega) and subsequently subcloned into the vector pBBR1MCS-5.

The vectors carrying the corresponding genes were introduced by conjugation into the PHA negative strain *P. putida* GPp104, following a modified protocol from Friedrich et al. [16]. Briefly, cell cultures were grown until exponential phase. Recipient and donor cells were mixed on a 1:1 ratio. Afterwards 1 ml of the mixture was centrifuged; the pellet was resuspended in 100 µl of 10 mM MgSO_4_ and put onto a sterile filter (0.22 µm, Millipore) over a LB agar plate at 30°C for 18 h. Subsequently, cells were washed off of the filter with 1 ml of 10 mM MgSO_4_, collected by centrifugation and plated on 0.5NE2 supplemented with 0.25% sodium octanoate and the adequate antibiotic concentration.

Cells from the transconjugant colonies were subjected to Nile Blue staining [17] in order to qualitatively analyze their ability to produced PHA. Quantitative determinations of PHA were also performed from lyophilized cells subjected to methanolysis and hot chloroform extraction. Methyl ester derivatives were analyzed by gas chromatography as previously described [18]. The PHA content was expressed as percentage of cell dry weight (CDW).

### Quantitative Real Time RT-PCR

Total RNA was extracted from pellets of late exponential phase cultures (OD_600_≈1.8) grown under PHA accumulation conditions as described earlier, using the Total RNA extraction kit (Real Biotech Corporation) according to manufacturer's indications. The RNA samples were subsequently treated with DNase I and the cDNA was synthesized using random hexamers (Promega) and AMV retrotranscriptase according to the manufacturer's instructions. Total RNA was quantified using a Nanodrop 2000 Spectrophotometer (Thermo Scientific). RT qPCR amplification of the cDNAs was performed in a LightCycler (DNA Engine M.J. Research) using Hot Firepol EvaGreen qPCR Mix Plus (no ROX, Solis Biodyne), plus 0.25 µM of the following forward and reverse primers corresponding to *phbC*, *phaC1* and *phaC2,* respectively: rtphbCup/low, rtphaC1up/low and rtphaC2up/low, (Table 2). In addition, primers rt16Sup/low were used for amplification of 16S rRNA gene, that was used for normalization of the *pha* genes expression levels. The cycling parameters used were: initial denaturation at 95°C for 5 min; 95°C for 15 s, 40 cycles of 56.4°C for 15 s, and a final extension step at 72°C for 5 min. The cDNA from three independent bacterial cultures grown with 0.25% sodium octanoate and 0.27% of glucose as carbon source were used for amplification.

Quantification of the fluorescence values obtained after amplification of the cDNAs was performed using a calibration curve as previously described [19]. Serial dilutions of genomic DNA of *P. extremaustralis* (0.014 ng to 1.65 ng) were used as template. Genomic DNA extraction was performed with the DNeasy Tissue Kit (Qiagen) following the manufacturer's instructions.

### Isolation of native PHA granules

Cultures of *P. extremaustralis* grown at 30°C in 0.5NE2 medium containing sodium octanoate were used for granule isolation. Exponential and stationary phase cultures were collected after 8 and 24 h of incubation, respectively. In addition, for carbon starvation, 24 h cultures were centrifuged, washed, resuspended in the same culture medium without carbon sources, and incubated for 5 h at 30°C. PHA granules were isolated as previously described in several works [20]. Cell extracts, obtained after disruption of cells through French press, were loaded into a glycerol gradient (87% v/v and 50% v/v). After ultracentrifugation, the granule layer found between the two glycerol solutions was reloaded on top of a second glycerol gradient (87%v/v, 80%v/v, 60% v/v and 40%v/v). Finally, the layer between the 60% and the 80% glycerol solutions was collected and subjected to overnight dialysis in 50 mM cold Tris-HCl buffer. The protein concentration was determined as described by Lowry [21]. The native granule extracts were analyzed by SDS-polyacrylamide gel electrophoresis (SDS-PAGE) [22] and stained with Coomasie Blue R-250 [23]. The major bands were excised from the gel and identified by high resolution mass spectrometry at the Laboratory of Proteomics (Institute of Biotechnology, UNAM, Mexico). Briefly, samples were reduced, alkylated, digested with trypsin and applied into a LC- MS system consisting of a micro flow Accela (Thermo - Ficher Co.) with “spliter” (1/20) coupled with a mass spectrometer LTQ- Orbitrap Velos (Thermo - Ficher Co.) with nano- electrospray ionization (ESI) source. Database search was carried out using Mascot and Protein Prospector programs.

### Sequences accession number

The sequences have been deposited in EMBL database under accession number AHIP01000001-AHIP01000135.

### Statistical analysis

For statistical analysis, three parallel experiments were conducted and data represent mean ± SD. Student's two-tailed t-test with confidence level >95% (P<0.05) was performed to determine the significance of differences.

## Results

### Characterization of genes involved in PHA metabolism in *Pseudomonas extremaustralis*

*Pseudomonas extremaustralis* is able to accumulate PHB (Table 3). Previous studies allowed us the identification of the gene cluster (*phaRBAC*), involved in PHB production, located within a genomic island [8]. Analysis of PHA production in *P. extremaustralis* showed that this bacterium is also able to produce low quantities of mclPHA during growth with 0.25% sodium octanoate or 3% glucose as carbon sources (Table 3). Since most of the bacteria belonging to the *Pseudomonas* genus have a conserved cluster of genes involved in mclPHA production, we searched for this cluster of genes in the genome sequence of *P. extremaustralis*. We found a mclPHA gene cluster comprised by two different genes encoding class II synthases, *phaC1* and *phaC2* (locus_tag: PE143B_0104515 and PE143B_0104505), which were separated by the *phaZ* gene (locus_tag: PE143B_0104510), encoding a putative intracellular PHA depolymerase. Inspection of the intergenic region between *phaC1* and *phaZ* revealed the presence of a hairpin structure of 18 bp (ΔG =  -37kcal), consisting of a palindromic sequence that displays dyad symmetry. This sequence was similar to the previously found in *P. corrugata* 388 and *P. putida* GPo1, which acts as a transcription terminator in both species [24], [25], suggesting a similar role in *P. extremaustralis*. A putative transcriptional regulator, *phaD* (locus_tag: PE143B_0104500), was found downstream of *phaC2.*

**Table 3:** Production of PHA in *P. extremaustralis* and recombinants *P. putida* GPp104.

Strain	Carbon source	Time of culture (h)	PHB content (wt% of CDW)	PHA content (PHHX+ PHO) (wt% of CDW)
*P. extremaustralis* 14-3b	0.25% octanoate	8	13.60±1.40	3.40±0.30
	0.25% octanoate	24	35.80±3.60	1.05±0.10
	0.27% glucose	8	0.37±0.04	ND
	0.27% glucose	24	1.30±0.10	ND
	3% glucose	24	12.20±1.00	0.26±0.02
*P. putida* GPp104 pMHZ59	0.25% octanoate	24	2.60±0.30	28.00±0.80
*P. putida* GPp104 pMMic2	0.25% octanoate	24	0.10±0.01	9.60±1.00
*P. putida* GPp104 pMHZ59	2% glucose	24	0.60±0.01	6.10±0.82
*P. putida* GPp104 pMMic2	2% glucose	24	0.27±0.03	2.66±0.33
*P. putida* GPp104 pBBR1MCS-5	0.25% octanoate	24	ND	ND
*P. putida* GPp104 pBBR1MCS-5	2% glucose	24	ND	ND

Cultures were grown in 0.5NE2 with the indicate carbon source.

ND =  no detected, PHHX: polyhydroxyhexanoate, PHO: polyhydroxyoctanoate.

**Figure 1:**
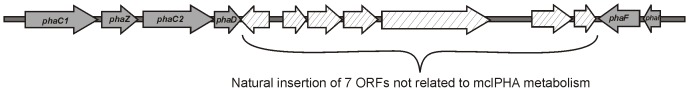
Genetic organization of the mclPHA gene cluster in *P. extremaustralis.* mclPHA genes belonging to *γ Proteobacteria* (smooth arrows). Natural insertion containing 7 ORFs related to *β Proteobacteria* between the genes *phaD* and *phaF* (broken arrows). Arrows indicate the direction of gene transcription and the relative size of each ORF. From left to right: *phaC1*(1680 bp), *phaZ* (846 bp), *phaC2* (1683 bp), *phaD* (621 bp), followed by genes encoding a LuxR family DNA binding response regulator (642 bp), a putative fimbrial subunit (579 bp), a bacterial pili assembly chaperone (780 bp), a pili assembly chaperone (759 bp), an outer membrane usher protein FimD (2505 bp), a putative fimbrial protein (945 bp) and a Pi fimbriae major subunit (528 bp), not related to PHA metabolism, and *phaF* (927 bp) and *phaI* (423 bp).

The availability of the genome sequence of *P. extremaustralis* allowed us to perform a more extensive analysis of the genes involved in mclPHA metabolism. In addition to the *phaC1ZC2D* gene cluster, two genes, *phaF* and *phaI* (locus_tag: PE143B_0104460 and PE143B_0104455), encoding phasin like proteins were identified. These genes were not located immediately downstream *phaD*, as observed in other *Pseudomonas* species [26], due to the presence of a natural insertion containing seven ORFs (locus_tag: PE143B_0104495, PE143B_0104490, PE143B_0104485, PE143B_0104480, PE143B_0104475, PE143B_0104470, PE143B_0104465) not related to PHA metabolism (Fig. 1). Some of these ORFs encode putative proteins related to *pili* and *fimbriae* similar to those belonging to β-Proteobacteria, like the P *pilus* assembly protein pilin FimA, a bacterial pili assembly chaperone and the outer membrane usher protein FimD. The deduced amino acid sequences of PhaC1, PhaZ, PhaC2, PhaD, PhaF and PhaI of *P. extremaustralis* showed a conserved size and high similarity with the corresponding proteins of other *Pseudomonas* species. The highest similarity was found with *P. fluorescens* SBW25 [27] sharing 98%, 78%, 96%, 95%, 94% and 75% of identity with PhaC1, PhaZ, PhaC2, PhaD, PhaF and PhaI, respectively. The aminoacids involved in the catalytic activity (Cys 296, Asp 452 and His 480) of the class II synthases [28] and other conserved amino acid (Trp 398 and Cys 431) were present in PhaC1 and PhaC2 of *P. extremaustralis.*

Complementation analyses of the genes encoding the class II PHA synthase genes were performed in order to determine if the identified *phaC1* and *phaC2* were functionally active. Plasmids pMHZ59 and pMMic2 containing *phaC1* and *phaC2*, respectively, restored PHA production in *P. putida* GPp104 (a derivative of *P. putida* KT2442 unable to produce PHAs), during growth with sodium octanoate or glucose as carbon sources (Table 3). Both class II PHA synthases were able to synthesize mainly mclPHA, however PhaC1 was also able to produce low quantities of PHB (around 3% of the cell dry weight) when the bacterium was grown with sodium octanoate as carbon source (Table 3). According to these results, both class II PHA synthases are functionally active in *P. extremaustralis.*

Further bioinformatic analysis of the genome of *P. extremaustralis*, allowed the identification of a cluster of 4078 bp, that was named *phbFPX*, presumably associated with PHB metabolism. The *phbFPX* cluster was located in the proximity (4000 bp of distance) of the previously identified *phaRBAC* cluster [8] involved in PHB biosynthesis. The genes included in the *phbFPX* cluster showed high similarity with sequences belonging to β-Proteobacteria, indicating that they were probably acquired by horizontal gene transfer. Prediction of genomic islands using the Island Viewer Program indicates that both clusters (*phaRBAC* and *phbFPX*) could belong to the same genomic island. The bioinformatic analysis of the cluster *phbFPX* (locus_tag: PE143B_0105770, PE143B_0105765, PE143B_0105760, respectively) revealed the presence of two genes encoding granule associated proteins (GAPs), named *phbF* and *phbP*, as well as an ORF that was named *phbX.* The deduced aminoacid sequences of PhbF and PhbP showed 100% identity with proteins of *Pseudomonas stutzeri* A1501 and between 60 and 76% identity with GAPs (phasin like or regulatory proteins) belonging to *Azotobacter* spp. The protein PhbX showed 97% identity with a protein annotated as PHA synthase in the *P. stutzeri* A1501 genome [29] and 62% identity with a putative PHA depolymerase of *Azotobacter vinelandii* DJ [30]. PhbX of *P. extremaustralis* showed an alfa-beta hydrolase domain as described for PHA synthases and depolymerases. However, the key amino acids involved in the functionality of PHA synthases could not be found in this protein and we were not able to complement the PHA negative strain *P. putida* GPp104 with a plasmid carrying *phbX* (data not shown). Likewise, the lipase box (G-X_1_-S-X_2_-G) present in many PHA depolymerases [31], could not be found in PhbX.

Based on the fact that several gene clusters involved in PHAs metabolism are present in the genome of *P. extremaustralis,* and in order to avoid confusion, the cluster involved in PHB metabolism that was previously named *phaRBAC* [7], [8] is now renamed *phbRBAC.*

### Expression of genes encoding PHA synthases

**Figure 2:**
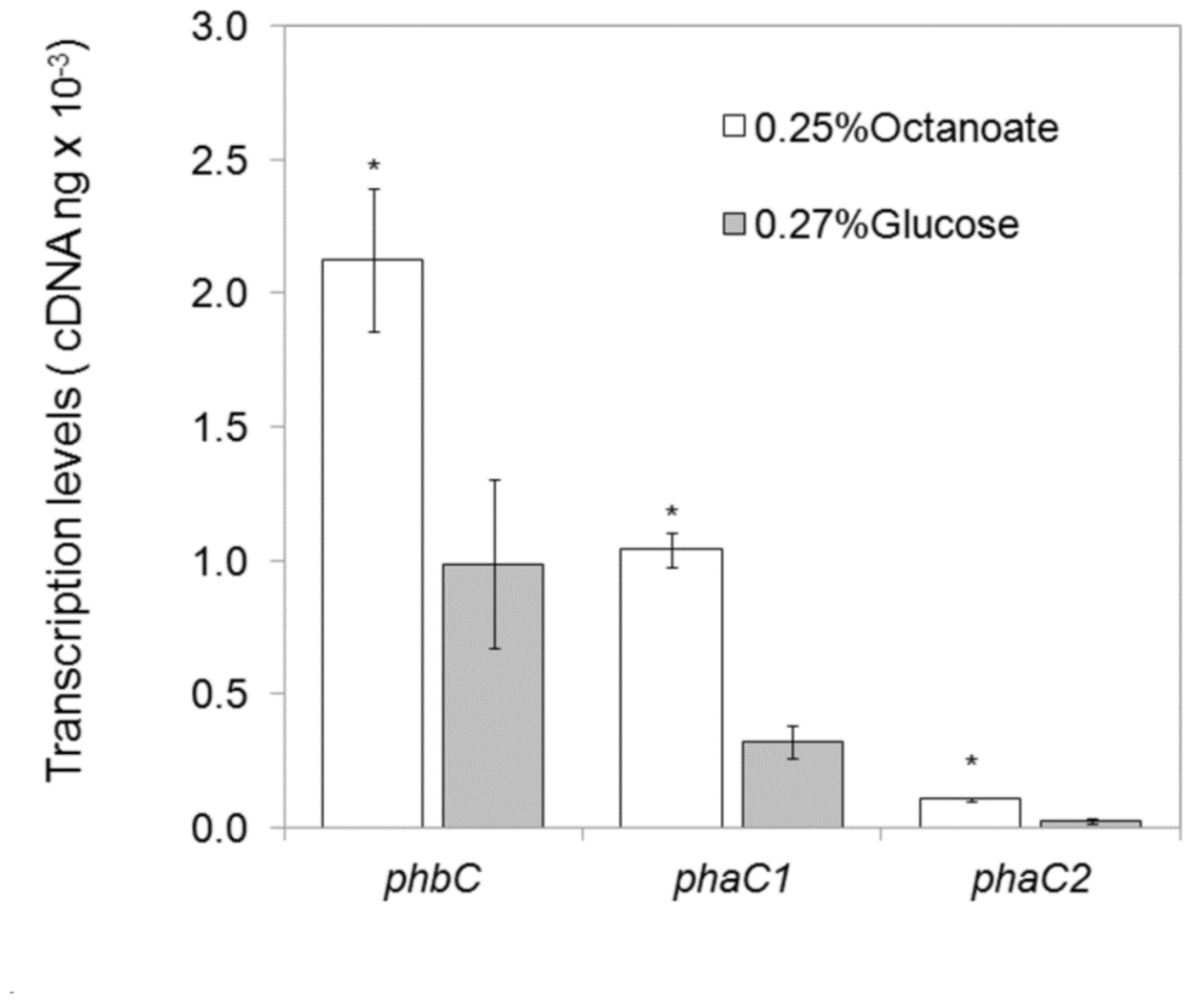
Analysis of the expression of *phbC, phaC1* and *phaC2.* qRT-PCR assays were performed with cells grown on 0.5NE2 supplemented with 0.25% sodium octanoate or 0.27% glucose until late exponential phase. Results are the average ± standard deviations from three independent cultures.

In order to understand why *P. extremaustralis* produces mainly PHB in spite of having genes encoding functional mclPHA synthases, we analyzed the expression of the three different PHA synthases present in the genome of this strain. The expression levels of the three PHA synthases genes were compared using RNA extracted from late exponential phase cultures (8h of growth, OD_600_≈1.8) grown under two different PHA accumulation conditions: 0.25% sodium octanoate (high PHA accumulation) or 0.27% glucose (low PHA accumulation). Cultures grown with sodium octanoate accumulated around 14% of PHB, while cells grown with glucose accumulated less than 1% of PHB. Levels of mclPHAs, polyhydroxyoctanoate (PHO) plus polyhydroxyhexanoate (PHHx) were less than 4% or undetectable, when bacteria were grown with sodium octanoate or glucose, respectively (Table 3). The expression of all the PHA synthases genes, *phbC, phaC1* and *phaC2*, was higher in cultures supplemented with octanoate. The level of expression of *phbC* compared with *phaC1* and *phaC2,* was significantly higher in both culture conditions (P<0.005, Fig. 2). The transcript levels of the class I PHA synthase, *phbC*, were 2 and 3 times higher than the transcript levels of *phaC1,* and 19 and 35 times higher than *phaC2* when the cells were cultivated with octanoate and glucose, respectively (Fig. 2). Finally, the expression level of *phaC1* was 9 and 11 times higher than the expression level of *phaC2* when bacteria were grown with octanoate and glucose, respectively.

### Analysis of the proteins associated to the PHA granules

**Figure 3:**
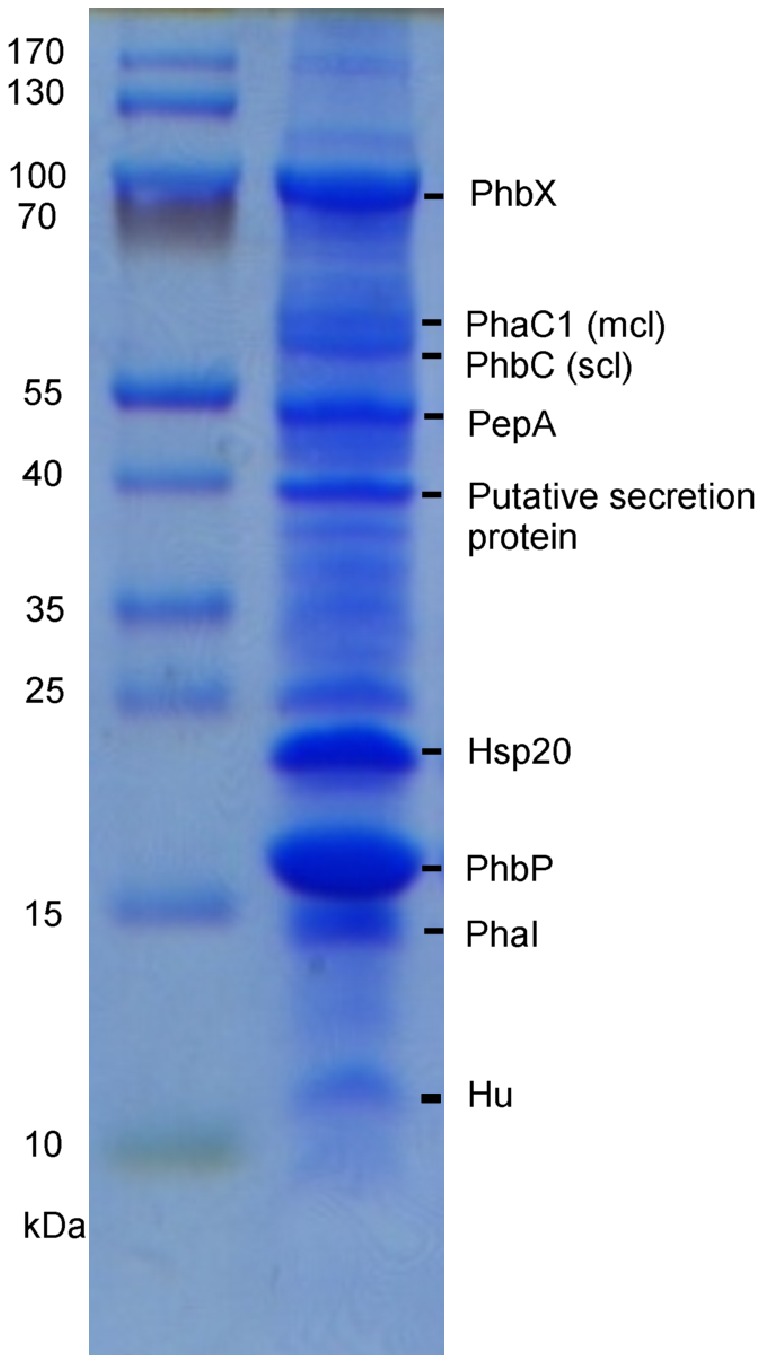
Proteins associated to the PHA granules of *P. extremaustralis.* Granules were obtained from cells grown under PHA accumulation conditions. Proteins were separated by SDS-PAGE, stained with Coomassie brilliant blue and subsequently identified by peptide fingerprint analysis as described in Materials and Methods. M, molecular mass standard.

To further investigate the expression and functionality of *P. extremaustralis* PHB and PHA synthases and to identify the proteins bound to the PHA granules in this bacterium, we carried out the isolation of the PHA granules and the analysis of the major proteins bound to these granules from cultures grown until exponential or stationary phase under different polymer accumulation conditions, using sodium octanoate or glucose as carbon sources. The composition of the proteins bound to the PHA granules was also analyzed from cells subjected to 5h of carbon starvation. SDS-PAGE of PHA granules, extracted from cells grown under different conditions, displayed a similar qualitative pattern of proteins (data not shown). For that reason, only the proteins obtained from granules extracted from 24 h-cultures, grown with sodium octanoate, were identified. Based on the molecular weight of the proteins normally found in PHA granules, and their relative amount in the SDS-PAGE (Fig. 3), nine bands were excised for the gel and subsequent identified by peptide fingerprint analysis (Table 4). Several proteins directly involved in PHA metabolism, such as the synthases PhbC and PhaC1, the phasin like protein PhaI and the phasin like protein PhbP, were identified among the granule bound proteins. PhbP represented the major band (Fig 3). In addition, PhbX, encoded by an ORF located downstream of *phbP*, was also found in the granules. Another four proteins, not known to be related to PHA metabolism, were also found: the cytosolic aminopeptidase PepA, a possible secretion protein, the heat shock protein Hsp20 and the DNA binding protein HU (Table 4).

**Table 4:** Proteins detected in PHA granules of *P. extremaustralis* by proteome analysis.

Protein	MW (kDa)	Locus_tag	Annotated function
PhbX	93.50	PE143B_0105760	Poly(3-hydroxyalkanoate) synthase
PhbC	64.15	PE143B_0105720	Poly(R)-hydroxyalkanoic acid synthase
PhaC1	62.58	PE143B_0104515	Poly(R)-hydroxyalkanoic acid synthase
PhbP	19.88	PE143B_0105765	Phasin
PhaI	15.31	PE143B_0104455	Putative polyhydroxyalkanoate biosynthesis-like protein
Aminopeptidase	52.42	PE143B_0109130	Cytosol aminopeptidase. COG0260 Leucyl aminopeptidase
Secretion protein	38.36	PE143B_0105715	Glycoside hydrolase family 43. Multidrug resistance efflux pump. COG0845 Membrane-fusion protein
Heat-shock protein	20.45	PE143B_0103035	Molecular chaperone Hsp20. COG0071 Molecular chaperone (small heat shock protein)
DNA binding protein HU	9.80	PE143B_0124435	Integration host factor. COG0776 Bacterial nucleoid DNA-binding protein

The annotated functions of the identified proteins, the locus tags of the coding genes and the calculated molecular masses (MW) are shown.

## Discussion

The analysis of the genome of *Pseudomonas extremaustralis* revealed the presence of different clusters of genes involved in PHA metabolism. Previous studies allowed us to determine that the cluster *phbRBAC*, involved in PHB metabolism, was located within a genomic island in *P. extremaustralis* [7], [8]. The genes belonging to this cluster have different origins, indicating that they were acquired by horizontal transfer, probably derived from *Burkholderiales* [8]. It has also been demonstrated that these genes confer adaptability to different stress conditions [32], [33].

In this work, an additional gene cluster, *phbFPX*, was found located 4000 bp upstream of the previously described *phbRBAC* [7]. This gene cluster showed similarity with *phb* genes found in diverse *Proteobacteria*, including *Burkholderiales* species, *Azotobacter vinelandii* and *P. stutzeri* A1501, suggesting the acquisition of these genes by horizontal transfer events. Interestingly, the region that includes both PHB gene clusters of *P. extremaustralis* showed high similarity with a genomic island with similar characteristics present in *P. stutzeri* A1501.

Besides, *phbFPX* and *phbRBAC,* the gene cluster *phaC1ZC2D*, identified as belonging to the *Pseudomonas* core genome, was found in *P. extremaustralis*. In several studied *Pseudomonas* spp., the phasin encoding genes, *phaF* and *phaI*, are located immediately downstream of *phaD* [24], [34], [35]. However, in *P. extremaustralis*, seven ORFs encoding proteins involved in pili and fimbriae assembly with similarity to *β-Proteobacteria* were found between *phaD* and *phaF,* suggesting the occurrence of an insertion in this region. This finding provides further evidence about the existence of genetic elements of diverse origin in the genome of *P. extremaustralis*.

PHA synthases are the enzymes responsible for PHA biosynthesis. Both class II synthases, PhaC1 and PhaC2, were able to restore the ability to synthesize mclPHA in a PHA minus strain of *P. putida*. PhbX, a putative synthase encoded in the *phbFPX* cluster, did not confer to *P. putida* GPp104 the capacity to synthesize PHAs, thus its role in PHA metabolism remains to be investigated.

The analysis of the expression of the genes *phbC* (encoding the PHB synthase) and *phaC1* and *phaC2* (encoding mclPHA synthases) of *P. extremaustralis* demonstrated that these three PHA synthases are expressed, but at different levels. When *P. extremaustralis* was cultivated with sodium octanoate as carbon source, the expression levels of *phbC, phaC1* and *phaC2* were significantly higher that when the cells were grown with glucose. The observed differences in the expression levels of the genes encoding PHA synthases correlated with a higher polymer production when *P. extremaustralis* was cultivated with sodium octanoate. In agreement with our results, RT-qPCR studies and analysis of the activity of the promoter regions of *phaC1* and *phaC2* conducted with different strains of *P. corrugata* and *P. putida* KT2442 showed higher expression levels for *phaC1* in comparison with *phaC2* when the cells were grown with octanoate as carbon source [36], [19]. The higher expression level of *phbC* in comparison with *phaC1* and *phaC2* could account for the higher percentage of sclPHA in comparison with mclPHA in *P. extremaustralis*. The type strain of *P. extremaustralis*, 14-3^T^, is a natural *phaA* mutant able to produce PHB from octanoate but not from glucose [6], [7] showing the capability of class I synthase to use monomers derived from fatty acids metabolism. In line with these results, strain 14-3b showed higher PHB production when grown in octanoate compared to glucose, indicating a high ability to use substrates derived from fatty acids for PHB biosynthesis. This fact, added to the higher expression of class I synthase, could result in a strong competence for substrate availability between PHB and mclPHA synthases.

The RT-qPCR assays of *pha* genes in *P. extremaustralis* agreed with the results obtained by the analysis of the granule associated proteins, in which PHA synthases involved in the synthesis of scl and mclPHAs, PhbC and PhaC1, were detected. In concordance with the low expression levels of *phaC2* in the RT-qPCR experiments, this protein was not detected in the PHA granules. In line with these results, PhaC2 was also not detected in PHA granules of *P. putida* GPo1, while a band of 60 kDa, corresponding to PhaC1 was identified [37]. The analysis of the proteins bound to the PHA granule in *P. extremaustralis* allowed us to identify a major band belonging to PhbP, protein directly associated with PHB production as the main phasin-like-protein, as well as the phasin PhaI, involved in mclPHA production. Analysis of the phasin-like-proteins bound to PHB granules showed that PhaP1 in *Ralstonia eutropha* and *Herbaspirillum seropedicae*, as well as PhaP in *Azotobacter* sp. FA8, proteins homologous to PhbP of *P. extremaustralis,* were found as the main GAPs [38], [9], [40]; whilst in mclPHA granules produced by *P. oleovorans* GPo1 the main GAP was PhaI [24]. In addition, we also found several proteins not directly related to PHA metabolism associated to the PHA granules, such as a cytosolic aminopeptidase. In PHA granules extracted from *P. putida* GPo1 a leucine aminopeptidase, with a putative role in the turnover of granule-associated proteins, was also detected [41]. This leucine aminopeptidase is similar to PepA of *E. coli* and PhpA of *P. aeruginosa*. The cytosolic aminopeptidase found in the PHA granules of *P. extremaustralis* showed 55% and 81% similarity to PepA of *E. coli* and PhpA of *P. aeruginosa,* respectively. In addition, in the PHB producers *R. eutropha* and *H. seropedicae* a leucyl aminopeptidase was also found [42], [39]. The DNA binding protein HU was also found associated to the PHA granules in *P. extremaustralis.* The role of this protein in the granules is not clear, but the presence of other histone like proteins, not directly related to metabolism, associated to the granules has been previously reported [39]. Nucleoid DNA-binding proteins involved in PHA metabolism have been described in both scl and mcl PHA producers. The proteins PhaM of the PHB producer *R. eutropha*, and PhaF found in the mcl-PHA-accumulating *P. putida*, interact with the DNA and the nucleoid ensuring equitable segregation of granules in daughter cells during cell division [43], [35].

The heat shock protein Hsp20, has also been found attached to PHA granules in *P. extremaustralis*. No heat shock proteins have been found in granules of native PHA producers, but in recombinant strains of *E. coli* carrying the genes for PHB production, an increase in the heat shock proteins DnaK and IbpAB has been observed associated with polymer granules [44], [45]. Moreover, DnaK and GroEL were detected in PhaP1 mutants of *R. eutropha* [46]. A putative secretion protein belonging to the HlyD family, with high percentage of identity to Type I antifreeze proteins, was also found attached to the PHA granules of *P. extremaustralis*. This protein presents a high content of alanine residues, characteristic of this kind of proteins [8]. The gene encoding this secretion protein is located next to the *phbC* gene, encoding the PHB synthase. A role of this protein in PHB metabolism remains to be investigated.

Due to the presence of the machinery to produce both scl and mclPHA in *P. extremaustralis*, we cannot determine if the identified granule-bound proteins proceed from a mixture of two types of granules composed only by one kind of polymer (PHB or mclPHA), or a single type of granule containing a mix of PHB and mcl-PHA. However, according to the polymer content data, the composition of the granules was mainly PHB.

Metabolism of mclPHAs in other *Pseudomonas* spp. has a complex regulation, which is controlled at the enzymatic level by cofactor inhibition and metabolite availability; at transcriptional level by specific and global transcriptional regulatory factors; and at translational level by global post-transcriptional regulators [47]. Up to now, only few reports have shown the presence of genes associated with the synthesis of scl and mclPHAs in the same organism [48], [49]. For this reason, the physiological and regulatory mechanisms that govern the production of both types of polymers in the same bacterium are still poorly understood. In this paper, we propose that the differential expression of PHB and PHA synthases may account for the high production of PHB in *P. extremaustralis*. An interesting finding of this work is that the PHB synthase, *phbC,* presumably acquired by horizontal transfer, showed higher expression in comparison with the Pseudomonads own mclPHA synthases, conferring the ability to produce high amounts of PHB in *P. extremaustralis*.

Finally, the study of the genes involved in PHA metabolism in *P. extremaustralis* could be useful for biotechnological purposes in order to achieve the production of different PHAs with varied elastomeric properties.
